# Effects of Intranasal Administration of Oxytocin and Vasopressin on Social Cognition and Potential Routes and Mechanisms of Action

**DOI:** 10.3390/pharmaceutics14020323

**Published:** 2022-01-29

**Authors:** Shuxia Yao, Keith Maurice Kendrick

**Affiliations:** The Clinical Hospital of Chengdu Brain Science Institute, MOE Key Lab for Neuroinformation, University of Electronic Science and Technology of China, Chengdu 611731, China

**Keywords:** intranasal administration, oxytocin, vasopressin, social cognition, blood–brain barrier, vagus, G protein-coupled receptors

## Abstract

Acute and chronic administration of intranasal oxytocin and vasopressin have been extensively utilized in both animal models and human preclinical and clinical studies over the last few decades to modulate various aspects of social cognition and their underlying neural mechanisms, although effects are not always consistent. The use of an intranasal route of administration is largely driven by evidence that it permits neuropeptides to penetrate directly into the brain by circumventing the blood–brain barrier, which has been considered relatively impermeable to them. However, this interpretation has been the subject of considerable debate. In this review, we will focus on research in both animal models and humans, which investigates the different potential routes via which these intranasally administered neuropeptides may be producing their various effects on social cognition. We will also consider the contribution of different methods of intranasal application and additionally the importance of dose magnitude and frequency for influencing G protein-coupled receptor signaling and subsequent functional outcomes. Overall, we conclude that while some functional effects of intranasal oxytocin and vasopressin in the domain of social cognition may result from direct penetration into the brain following intranasal administration, others may be contributed by the neuropeptides either entering the peripheral circulation and crossing the blood–brain barrier and/or producing vagal stimulation via peripheral receptors. Furthermore, to complicate matters, functional effects via these routes may differ, and both dose magnitude and frequency can produce very different functional outcomes and therefore need to be optimized to produce desired effects.

## 1. Introduction

Over the last few decades, acute and chronic administration of intranasal oxytocin (OXT) and vasopressin (AVP) has been extensively utilized in both animal and human studies to modulate various aspects of social cognition and their underlying neural mechanisms. Functional effects of these neuropeptides vary from basic emotional recognition and social reward to complex social behaviors such as altruistic behavior and moral decision making, although some effects have been inconsistent [1,2,3]. Given a lower systemic exposure and a more efficient targeting, intranasal delivery can be an optimal strategy for a number of the central nervous system-acting drugs with fewer side effects (see [4,5,6]). Both OXT and AVP administered intranasally are considered to have great therapeutic potential for mental disorders characterized by deficits in social cognition, motivation, and affective processing, such as autism, anxiety, depression, and schizophrenia (see [1,7]) and have widespread influences via their receptors in the brain regions involved [8,9]. For example, in the context of autism, studies have demonstrated that intranasal OXT increases attention towards social cues, such as the eyes of faces [10,11], and behavioral and neural responses to social reward [12,13,14]. Several clinical trials have also reported improved social symptoms following chronic treatment with intranasal OXT [15,16,17] or AVP [18] in young children and with no reports of any serious side effects. A systematic review of 38 randomized, controlled trials using single doses of intranasal OXT conducted between 1990 and 2010 also did not find any evidence for significant side effects [19].

The blood–brain barrier (BBB) has long been recognized as a significant impediment for therapeutic drug development [20], and an intranasal route of administration has increasingly been proposed as a way of circumventing this problem through by-passing it (see [21]). The adoption of an intranasal route of administration for OXT and AVP also occurred as a result of animal model studies primarily reporting effects on social behaviors and corresponding neural circuitry following direct infusions into the brain and evidence that the blood–brain barrier was relatively impermeable to them [22,23]. While a number of studies had indicated that the intranasal route could be effective in delivering drugs or peptides directly to the brain via the olfactory and trigeminal nerves (see [5,21]) there has been considerable controversy concerning whether this is the route whereby OXT or AVP produce their observed functional effects (e.g., [24,25]). However, a growing number of studies in both animal models and humans have provided evidence that these neuropeptides can indeed enter directly into the brain via the olfactory and trigeminal nerve projections [4,26].

The precise mechanism whereby neuropeptides such as OXT and AVP can enter the brain via the olfactory and trigeminal nerve routes is not fully established. There are several steps required before an intranasally administered substance can be delivered to the brain: it must first be transported across the olfactory or respiratory epithelial barriers in the nasal passages and then from the nasal mucosa to sites of brain entry via peripheral olfactory or trigeminal nerve components, and finally be transported from these initial brain entry sites to other regions within the brain (see [5,6] for extensive reviews of these transport mechanisms). Once substances reach the olfactory and trigeminal nerves, they can potentially be transported into the brain via intracellular (endocytosis and intraneuronal transport within olfactory sensory neurons and trigeminal ganglion cells) or extracellular (diffusion or convection within perivascular or lymphatic channels associated with the nerves and extended from the lamina propria into the brain). Transport via the intracellular route is somewhat slower than by the extracellular ones (again see [5,6] for extensive reviews). Given that studies using intranasal administration of labeled peptides, including OXT, have observed relatively fast transport into the brain, the current view is that extracellular convection (bulk flow) is the most likely mechanism for acute doses [5,27], although it is possible that the other potential intracellular and extracellular mechanisms might also contribute with chronic treatment.

The mechanisms whereby intranasal administration of OXT and AVP produce their functional effects are still a matter of debate, however, particularly given that in addition to them entering the brain directly, there is increasing evidence suggesting potential contributions via other peripherally mediated routes (see [28,29]). In this review, we will focus on research in both animal models and humans, which has investigated the different potential routes via which these intranasally administered neuropeptides may be producing their effects on social cognition and how we can distinguish between functional effects of intranasal administration mediated via these different routes. We will also consider the importance of dose magnitude and frequency for influencing G protein-coupled receptor signaling and subsequent functional outcomes and additionally the contribution of different methods of intranasal application.

## 2. Different Routes Whereby Intranasal Oxytocin and Vasopressin May Produce Functional Effects

**Figure 1:**
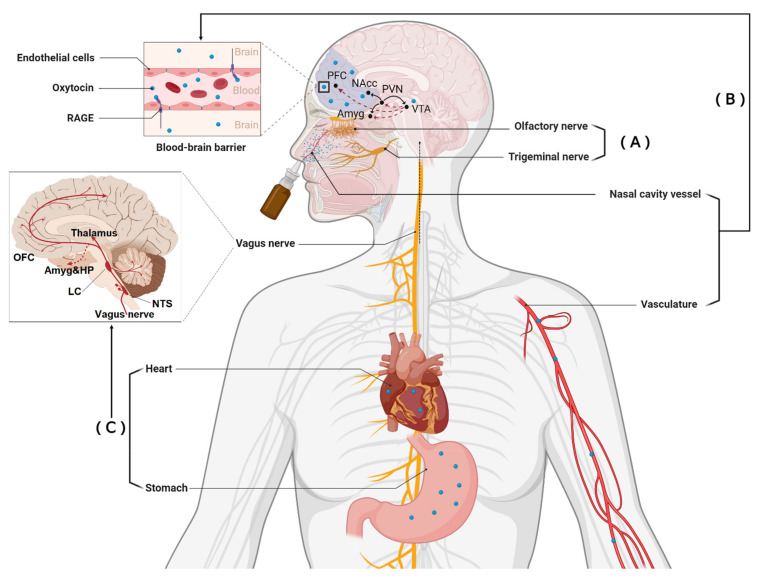
Routes of intranasally administered oxytocin (OXT) can influence brain function and its functional effects. (**A**) Direct entry into the brain from the back of nose via olfactory and trigeminal nerves. (**B**) Indirect entry into the brain from the peripheral circulation via binding to the receptor for advanced glycation end-products (RAGE). (**C**) Indirect brain modulation of peripheral OXT via the vagus by acting on receptors in peripheral organs (heart and gastrointestinal system). PFC: prefrontal cortex; NAcc: nucleus accumbens; PVN: paraventricular; Amyg: amygdala; VTA: ventral tegmental area; OFC: orbitofrontal cortex; HP: hippocampus; LC: locus coeruleus; NTS: nucleus tractus solitarius. Figure 1 is adapted from [31], Frontiers Media S.A., 2021.

Although researchers have mainly focused on demonstrating that intranasally administered OXT and AVP can produce functional effects via direct entry into the brain (for example, see [30] in the context of analgesic effects), they also enter the peripheral circulation via blood vessels in the nasal cavity. Additionally, some of the intranasally administered peptides can ‘drip down’ into the mouth cavity, where they can also be absorbed by blood vessels and ingested to produce effects via receptors in the gastrointestinal system. Thus, there are potentially multiple routes whereby intranasal administration of neuropeptides can produce functional effects (see Figure 1).

### 2.1. Direct Entry into the Brain

Increased concentrations of OXT in the cerebrospinal fluid (CSF) occur following intranasal administration in both rhesus macaques (using an intranasal mucosal atomization device (MAD) or nebulizer) [27,32]) and in both the AVP and OXT in humans [33,34]. While this in itself does not constitute proof that the neuropeptides have directly entered the brain, accumulating lines of evidence suggests that they can probably do so directly via the olfactory and trigeminal nerve projections [4,26] (see Figure 1). More specifically, based on approaches using labeled peptides, intranasally administered OXT has been shown to accumulate in brain regions such as the amygdala and hippocampus in rodents [35] or increase concentrations in regions with an OXT receptor (OXTR) using intracerebral microdialysis sampling methods [36,37]. A study using the measurement of regional cerebral blood flow (rCBF) changes has also reported activation of hippocampal and forebrain regions in the mouse characterized by high OXTR density following intranasal administration of OXT, although a divergent deactivation pattern was observed following intranasal administration of AVP [38]. A more recent study using intranasal administration of labeled OXT in rhesus macaques has also found measurable concentrations of labeled peptides in brain regions, including the orbitofrontal cortex (OFC) and striatum, which are targets of olfactory and trigeminal nerves projections (using an intranasal MAD), although not following intravenous infusion [39]. In humans, intranasal (using a standard nasal spray or a nebulizer) OXT produces changes in rCBF in reward networks (OFC and striatum) and additionally in emotional and salience processing networks (the insula, amygdala, and anterior cingulate gyrus) as well as visual processing (the right occipital gyrus, lingual and fusiform gyri) and default mode (medial frontal cortex, hippocampus) networks, peaking at around 39–51 min post-administration [40,41]. The functional effects of OXT administered via the intranasal route can be exerted via binding to widely distributed OXTR in the brain [9], with regions showing higher OXTR expression being more responsive to OXT modulation independent of the task type and gender [42]. Overall, these findings across species and using different methods provide compelling evidence that intranasally administered OXT and AVP can directly enter the brain, although they do not preclude the possibility that they can produce additional effects via peripherally mediated routes.

### 2.2. Indirect Entry into the Brain from the Peripheral Circulation

While, as already mentioned above, research on intranasal neuropeptide administration has primarily focused on functional effects produced via direct entry into the brain, the original pharmaceutical application for both synthetic OXT (Syntocinon nasal spray—Novartis) and AVP (Minirin—Ferring Pharmaceuticals) was to produce effects on peripheral organs via absorption into nasal blood vessels and entry into the peripheral circulation (i.e., originally intended to enhance lactation or to help control kidney function and urination). Thus, intranasal administration of OXT, either via a standard nasal spray or a nebulizer, increases concentrations in peripheral blood across species, although with a slower temporal profile (increases occur within 10–15 min proceedings of the end of administration) compared with intravenous administration (occurs immediately at the end of administration) [27,28,32,34,36,37,40,43]. Increased plasma concentrations of AVP have also been reported following both intranasal and intravenous administration [33,44]. Additionally, intranasally administered neuropeptides can “trickle-down” into the mouth, as indicated by high saliva OXT concentrations immediately and up to 7 h after administration using both the standard nasal spray and the breath-controlled nebulizer [45,46,47]. Thus, peptides entering the mouth can additionally be absorbed by blood vessels and subsequently enter the gastrointestinal system following ingestion. A few studies applying OXT via an oral route have reported increased blood concentrations following administration in both mice [48] and humans [28,49]. It should also be noted that after controlling blood vessel absorption in the mouth by applying OXT in the form of a capsule, after reaching the intestinal tract, it could still dramatically increase plasma OXT in mice pretreated with a proton pump inhibitor (omeprazole) to reduce degradation of the peptide by acid in the stomach [48].

While it had widely been assumed that the BBB was almost impermeable to OXT and AVP and that consequently, any effects on the brain and behavioral functions were unlikely to be influenced by increased concentrations in blood, this assumption is no longer tenable. A key recent finding from animal studies is that OXT in the peripheral blood can cross the BBB by binding to the receptor for advanced glycation end products (RAGE) [50]. The functional relevance of this was demonstrated by showing that whereas OXT can normally induce maternal bonding behavior following both central and peripheral administration, in RAGE-knockout mice, it only occurs following central administration [51]. Although it is unclear whether AVP can also cross the BBB this way, it seems likely that this kind of mechanism may permit other peptides from crossing it, although perhaps still in quite limited amounts. Additionally, there is a possibility that OXT and AVP may be able to cross the blood–cerebrospinal fluid barrier and/or enter the brain via the circumventricular organs [4].

Further indirect support for peripherally mediated effects of OXT has come from an increasing number of studies reporting functional effects when it is given by other peripheral administration routes that, unlike the intranasal route, would not permit direct entry into the brain (i.e., intravenous, intraperitoneal, or oral). For example, high doses of intraperitoneal-administered OXT can still modulate the social behaviors of mice, although findings are less consistent and differ in some respects compared to central administration [52,53]. In humans, orally (lingual spray) administered OXT has been reported to increase attention to social stimuli in an anti-saccade task in the same way as intranasal administration and at the same 24 IU dose [29]. Two early studies have also reported improvements in repetitive behaviors and social cognition in autistic individuals following slow intravenous OXT infusions [54,55]. In terms of altered neural activity in humans, intravenous administration of OXT has been found to induce a similar effect to the intranasal on decreases in amygdala perfusion at rest [40], although a previous study in rats did report some differences [56]. A paper using a small number of human subjects has also reported that a low dose of intranasal OXT altered amygdala responses to emotional faces, whereas an intravenous infusion did not [57]. Orally (lingual spray) administered OXT can increase both arousal and associated neural responses in the reward system and amygdala in response to face emotions, with enhanced responses in the reward system being partially mediated by increased blood OXT concentrations [28]. However, these effects were in contrast to an intranasal administration of the same dose, where amygdala responses and arousal were reduced, and no effects in the reward system were observed [58]. While less recent research has been conducted for AVP in terms of peripherally mediated effects, early research on memory-enhancing effects of the peptide also demonstrated some similarities between central and peripheral administration routes (see [59]).

## 3. Acting on Receptors in Peripheral Organs to Influence the Brain via the Vagus

In addition to peripheral OXT entering into the brain via binding to RAGE, peripheral OXT can influence brain function indirectly via the autonomic nervous system, particularly sympathetic and vagal pathways, by acting on its widespread receptors in peripheral organs (notably in the cardiac and gastrointestinal systems) [60]. There is also evidence for modulatory effects of AVP on autonomic function [61,62], with its receptors also distributed in cardiac and gastrointestinal systems [63,64]. Intranasal administration of both OXT and AVP thus could produce functional effects by influencing activity in widespread brainstem, limbic and cortical regions following peripherally mediated vagal stimulation (see [65]) and/or by evoking endogenous release of the peptides within the brain [66]. Subdiaphragmatic vagotomy (SDV) provides the possibility for causally isolating vagal-dependent OXT effects. Animal model studies have shown, for example, that inhibitory effects of peripherally (intravenously and intraperitoneally) administered OXT on methamphetamine self-administration and reinstatement and food intake can be prevented by SDV [67,68]. Although there is no direct evidence in humans, OXT is found to mediate human parasympathetic and sympathetic responses, including heart rate, heart rate variability, and pre-ejection period both at rest and during task-based paradigms [69,70,71,72,73] (but see [40]).

## 4. How Can We Distinguish between Functional Effects of Intranasal Administration Mediated via These Three Different Routes?

As reviewed above, functional effects of OXT can be exerted by direct entry into the brain via the olfactory and trigeminal nerves [4,26] see also [30]), indirect entry into the brain after binding to the RAGE [50,51], or by indirectly influence brain function via producing vagally mediated effects by acting on its receptors in peripheral organs [60]. Thus, functional effects of intranasal OXT previously observed can be contributed to by all three routes, and it is difficult to reliably distinguish between them based on currently available methodologies.

A straightforward, but coarse strategy, for distinguishing the contribution of these different routes to functional effects of intranasal AVP or OXT administration is by conducting direct comparisons based on the same paradigms. Findings in animal models have been somewhat inconsistent. In rats, for example, OXT concentrations in the brain were much higher following intranasal relative to intravenous administration, with >95% of OXT in the brain being directly transported from the nasal cavity. In contrast, blood concentrations were much higher following intravenous compared to intranasal administration, but only intranasal OXT produced an anxiolytic effect by decreasing corticosterone concentrations [74]. This suggests that the anxiolytic effect of intranasal OXT was only driven via direct entry to the brain via the olfactory and trigeminal nerve routes. On the other hand, a study comparing direct central (intracerebroventricular 1–10 μg) and peripheral (intraperitoneal 3–30 mg/kg) administration has revealed similar dose-dependent effects of OXT on increasing punished crossings in the four-plate test, a measure of anxiolytic-like effects. Furthermore, the anxiolytic effects of OXT administration via both routes could be blocked by receptor antagonists acting at a central but not peripheral level, supporting the view that peripherally administered OXT was crossing the BBB and producing similar effects on the brain as intracerebroventricular administration [52]. However, the same study reported route-specific effects of OXT on a social investigation in the elevated zero-maze test (only intracerebroventricular) and attenuation of stress-induced hypothermia (intraperitoneal). Another study found opposite effects of intracerebroventricular and intraperitoneal administration of OXT on stress-related behavior [53].

In humans, intranasal and intravenous administration of OXT can induce different profiles of plasma concentrations, with intravenous OXT reaching the peak level immediately after dosing whereas increased concentrations of intranasal OXT being observed 10–15 min following application [40,43]. These two administration routes also showed some dissociative functional effects with intranasal OXT dose-dependently (only the 8 IU dose via nebulizer), decreasing anger ratings of emotionally ambiguous faces [43], amygdala responses to emotional faces [57], and pupil diameter in response to different emotional faces [75]. On the other hand, intravenous OXT (1 IU) did not produce significant effects on these measures compared to the placebo group, although subject numbers were quite low (n = 16), and there were no significant differences between the effects of 8 IU intranasal OXT and intravenous OXT. Similar dissociative functional effects have also been reported for intranasal (20 IU) and intravenous administration of AVP (0.1, 0.025, and 1.5 IU) on auditory evoked potentials [44], although again, subject numbers were low (n = 15). A potential issue that may have contributed to inconsistent findings using intravenously administered OXT and AVP is that they are metabolized very quickly in the blood (3–5 min) [23,74] and possibly duration, as well as doses used, may influence the outcome. Thus, for example, a number of studies reporting no effects carried out using low-dose (0.025–1.5 IU) intravenous infusions over around 20 min [43,44,57,75], whereas two which did report effects carried them out using higher doses (infusion rate escalating up to 700 mL/h of 10 IU/L) over a period of 4 h [54,55]. Similar effects have also been found between intranasal and intravenous OT administration on decreasing amygdala perfusion at rest, whereby 40 IU vs. 10 IU (over 10 min) were used for intranasal and intravenous, respectively [40]. Intravenous infusions of these peptides may therefore not be the optimal way to try to reproduce the dynamics of peripheral effects of intranasal treatment.

Most recently, direct comparisons between intranasal and oral (lingual) administration have also been conducted in humans. One study demonstrated that orally 24 IU OXT increased neural activity of the reward system and amygdala in response to emotional faces, whereas 24 IU intranasal OXT had no effects on neural responses in the reward system and, in contrast, decreased the amygdala activation in response to emotional faces [28]. Furthermore, in this case, statistical comparisons between oral and intranasal effects showed that they were significantly different. However, in an emotional anti-saccade eye-tracking paradigm which measures both top-down and bottom-up attention, 24 IU intranasal or oral OXT both showed similar effects on weakening top-down attentional control of the instructed direction by increasing interference of social stimuli (emotional faces) in the anti-saccade condition, although the oral relative to the intranasal route, facilitated bottom-up attentional engagement towards emotional faces in the pro-saccade condition [29]. Thus, the studies utilizing peripheral administration (intravenous, intraperitoneal, and oral) routes compared with central or intranasal ones demonstrate both similar and different functional effects, indicating that both can contribute to circumstances where an administration route (such as intranasal) produces direct (penetration into the brain) and indirect (via increased peripheral concentrations either crossing the BBB into the brain or via vagal stimulation) effects.

It should be noted that at this point, it is difficult to disentangle whether OXT indirectly entering the brain via binding to RAGE or causing vagal stimulation are responsible for peripherally mediated effects. In this respect, animal models offer the greatest promise, with RAGE-knockout rodents providing a method for preventing functional effects of OXT crossing the BBB and influencing the brain, as already demonstrated in the context of maternal behavior and hyperactivity [51]. For determining vagally-driven effects, then again, animal models incorporating cutting the vagus will be informative (as in [67]), as well as determining whether functional effects can be blocked using receptor antagonists, which can only act peripherally (see [52]). Overall, though, the findings to date from both animal model and human studies comparing different routes of administration suggest that functional effects can be similar but can also show some route dependency and may, in some cases even, produce opposite effects. This, therefore, needs to be taken into account when interpreting effects produced via intranasal administration.

## 5. Characteristics of Oxytocin and Vasopressin Receptors

Both OXT and AVP have classical seven-transmembrane domain G protein-coupled receptors, with to date OXT having only a single identified receptor and AVP having three (V1, V2, and V3). Additionally, both OXT and AVP can also bind to each other’s receptors, although with a much-reduced affinity (around a 10-fold reduced affinity) [76].

### Effects of Dose Magnitude and Dose Frequency

Although OXT only has a single receptor, it recruits three different intracellular G protein-coupled pathways (Gq, Gi, and Go) in a concentration-dependent manner which can result in opposite effects on neuronal activity, with increasing OXT bioavailability shifting coupling away from the excitatory Gq-protein to the inhibitory Gi- or Go- ones [77]. It would appear that the positive effects of intranasal OXT on social cognition may be primarily via Gq-protein signaling. There is evidence for an inverted U-shaped dose-response curve for acute OXT (nasal spray) effects in humans in the context of social cognition, with higher doses having the opposite effects of lower ones [78]. A similar inverted U dose-response curve has been reported for rCBF responses to intranasal OXT [79], and in both cases may be the result of higher concentrations producing a shift away from recruiting Gq-protein coupled pathways towards Gi- or Go-protein ones. Dissociative functional effects have been reported between high and low doses of intranasal OXT (8 vs. 24 IU) and AVP (20 vs. 40 IU) in face processing tasks [43,57,75,80]. It has also been suggested that inverted U dose-response curves for responses to OXT may demonstrate sex differences in the context of social reward [81], and this possibility is further supported by a number of studies in humans showing opposite functional effects on the same intranasal OXT dose in males and females [82,83,84]. Additionally, G protein-coupled receptors are well known to exhibit desensitization, internalization, and subsequent down-regulation following exogenous treatment with agonist ligands [85], particularly the Gq-protein coupled sub-units of both the OXTR and AVPR1a receptors [77]. Chronic daily intranasal OXT treatment can therefore result in extensive OXTR and AVPR1a receptor down-regulation in the rodent brain [86,87]. Not only have animal model studies demonstrated that chronic intranasal dosing with OXT can lead to receptor down-regulation in the forebrain, but it can also result in impaired rather than enhanced social behavior [86,88] and increased rather than decreased anxiety [89]. In humans, we have shown that once-daily treatment with intranasal OXT leads to receptor desensitization with reduced neural responses to emotional faces and anxiolytic response to threatening stimuli, which occur after a single dose is abolished after five days of daily doses. On the other hand, both neural and behavioral effects are maintained or enhanced after five days by using a less frequent administration every other day to allow the receptor more time to recover from desensitization following each dose [58]. In a follow-up experiment, we replicated this effect by showing that only intranasal OXT administered every other day reduced neural responses to threatening stimuli after five days in individuals with high trait anxiety [90]. Thus, OXT that is given intranasally both at a too-high concentration or a too-great frequency can result in reduced or altered functional effects on social cognition either by producing greater effects on Gi- or Go-coupled signaling and/or by reducing effects via G-q coupled signaling as a result of internalization and down-regulation.

## 6. Influence of Different Types of Intranasal Administration Device

A variety of different methods have been used for the intranasal administration of OXT or AVP. In small animals, a small volume of liquid is deposited into the nostrils for the animal to inhale naturally [86,88]. In monkeys and humans, OXT and AVP are generally administered either using a spray bottle (usually 0.1 mL per individual spray) or using breath-controlled nebulizers [40,43,57,79,91,92,93]. It is generally proposed that the optimal strategy for uptake of intranasal peptides into the brain via the olfactory and trigeminal nerves is for the spray delivery device to deposit the peptides into the most posterior part of the nasal cavity, and this is considered to be achieved best using a nebulizer device which has the advantage of finer droplet sizes and being propelled more effectively into the posterior part of the nasal cavity [94,95]. It is not our intention to review the various different methods which can be used (see [94,95,96]) but rather if they achieve different quantitative or qualitative functional effects in the domain of social cognition.

The vast majority of intranasal OXT and AVP administration have used the standard nasal spray device, and relatively few have used the more efficient nebulizer type. One study in macaques has compared differential effects of the two devices for intranasal administration and demonstrated that while both produced similar increases in cerebrospinal concentrations 40 min after administration, the increases produced by the nebulizer were more robust. On the other hand, increased blood concentrations only occurred with the standard nasal spray device from 10–40 min after administration [91]. This would seem to suggest that the nebulizer administration technique may be slightly more efficient at getting the peptide directly into the brain but much less efficient at getting it into the peripheral circulation. In humans, the only comparisons to date we can make are in relation to the optimal doses for achieving the same functional effects during tasks with the two different administration devices. These studies have suggested different dose-response curves for amygdala responses to emotional faces, with the standard spray device demonstrating an optimal effect at 24 IU [78] compared to the nebulizer one at 8–10 IU [57,79,93] in males. As already mentioned above, it is possible that optimal doses may be different in females [81,83]. On the other hand, one study which has directly compared effects of intranasal OXT administered by conventional spray and nebulizer devices at the same relatively high dose (40 IU) has reported that they produce different patterns of rCBF changes in the brain, which might possibly reflect greater penetration into posterior regions of the brain by the nebulized dose [40]. However, since other doses were not used in this study, it remains possible that this may reflect different dose-response curves for the conventional spray and nebulizer-administered peptide. Indeed, in a subsequent study, the same group has reported different patterns of rCBF changes in amygdala subregions with 9, 18, and 36 IU doses of OXT administered using a nebulizer [79].

Although intranasal OXT studies using conventional and nebulizer administration devices have suggested that the latter can achieve similar functional effects at lower doses, to date for social cognition tasks, there is no evidence that at these optimal doses they differ in terms of the nature of functional effects produced, although there are still relatively few studies using nebulizer devices. Furthermore, in contrast to the study on monkeys mentioned above [91], a study in humans has reported that blood concentrations of OXT were significantly increased by both 8 IU and 24 IU doses of OXT administered using a nebulizer [43]. Similarly, the study reporting different patterns of rCBF changes in the brain after administration of 40 IU by conventional spray compared with a nebulizer reported similar profiles of increased OXT concentrations in the blood with the two methods [40]. Thus, despite the greater efficiency of nebulizers in administering OXT to the posterior part of the nasal cavity, and thereby more efficiently delivering the peptide directly into the brain via the olfactory and trigeminal nerves, it would appear that as with the conventional nasal spray devices, they also increase peripheral OXT concentrations. Therefore, it would seem that both the conventional spray devices and the nebulizers used to date could produce functional effects via OXT entering the brain directly and/or after entering the peripheral circulation and crossing the BBB or by stimulating the vagus. Possibly different nebulizers may be more efficient at only increasing OXT via direct entry into the brain and have minimal effects via the peripheral circulation, and this might lead to different functional effects being produced. For example, evidence suggests that different droplet size produced by different nebulizers influence their efficiency in depositing substances in the posterior region of the nasal cavity [97]. However, the presence of inverted U dose-response curves will still complicate matters.

## 7. Conclusion and Future Directions

In conclusion, evidence from both animal and human studies suggests that while functional effects of intranasally administered OXT and AVP in the domain of social cognition may result from direct penetration into the brain, observed effects may also be contributed to by the peptides either entering the peripheral circulation and crossing the BBB via binding to the RAGE and/or producing vagal stimulation via peripheral receptors. However, based on current methodologies, especially in humans, a distinction of the relative contributions of these different routes to observed functional effects is still unclear. Indeed, inconsistent findings from intranasal administration studies in the domain of social cognition may be influenced by the relative contributions of the different routes, especially given some evidence that there may be opposing functional effects of direct vs. peripheral routes [28,52,53]. Future studies applying different administration strategies should take possible contributions of different routes into consideration when interpreting findings. To further improve our understanding of a more precise mechanism underlying functional effects of the neuropeptides, future studies should also aim at developing innovative methods that can distinguish between functional effects of intranasal administration mediated via these different routes. Given that dose magnitude and frequency can also influence G protein-coupled receptor signaling and consequently produce very different functional outcomes, more studies are also needed to determine and optimize modulatory influences of dose magnitude and frequency.

## Data Availability

No new data are included in this review.
